# Psychometric properties of a new instrument for the measurement of the perceived quality of distance learning during the coronavirus disease 2019 (COVID-19) pandemic

**DOI:** 10.3389/fpsyg.2023.1169957

**Published:** 2023-08-07

**Authors:** Maria Rita Sergi, Laura Picconi, Aristide Saggino, Alessandra Fermani, Ramona Bongelli, Marco Tommasi

**Affiliations:** ^1^Department of Medicine and Aging Sciences, University of Chieti-Pescara, Chieti, Italy; ^2^Department of Education, Cultural Heritage and Tourism, University of Macerata, Macerata, Italy; ^3^Department of Political Science, Communication and International Relations, University of Macerata, Macerata, Italy

**Keywords:** COVID-19, anxiety, depression, perceived self efficacy, distance learning

## Abstract

**Introduction:**

The lockdown restrictions due to the COVID-19 pandemic forced many students to use distance learning. Few studies have examined the psychological effects of distance learning during the pandemic on university or on non-university students using a specific psychometric tool. The principal aim of this study was the construction and validation of a new psychometric tool, the Perceived Quality of Distance Learning (PQDL), to measure students’ appreciation and reaction to distance learning. The connection between anxiety, depression, perceived self-efficacy, and students’ perception of distance learning was analyzed to assess the nomological validity of the new scale.

**Method:**

The sample consists of 429 students who attended university or training courses. The factor structure of the new instrument was analyzed through Exploratory and Confirmatory Factor Analyses and its nomological validity was analyzed through regression analysis.

**Conclusion:**

The results showed that PQDL consists of two subscales: Distance Learning Organization and Cognitive-Emotive Reaction to Distance Learning. Higher student’s ability to organize and plan distance learning and higher student’s positive cognitive-emotive reaction to distance learning, higher student’s perceived quality of distance learning. Anxiety and depression scores were negatively correlated with students’ perceived quality of distance learning. Furthermore, students’ perceived emotional self-efficacy of negative emotions and perceived scholastic self-efficacy were positively correlated with students’ perceived quality of distance learning. These data indicate that PQDL is a reliable questionnaire to assess student’s perceived quality of distance learning.

## Introduction

Recent data showed that the Coronavirus disease 2019 (COVID-19) pandemic had a negative impact on the psychological well-being of different population samples due to restrictive measures (Gismero-González et al., 2020; Khoshaim et al., 2020; Paolini et al., 2020; Macdonald and Hülür, 2021; Partouche-Sebban et al., 2021). These included the halting of social and working activities, the reduction of social interactions, and a reduction in economic resources. These factors were associated with behavioral changes, such as insomnia or sleep disorders, concentration and attention difficulties, and low appetite (Bao et al., 2020; Holmes et al., 2020; Tommasi et al., 2020). In particular, the pandemic reduced people’s confidence in their ability to deal with everyday problems, as people’s level of uncertainty (Taylor, 2019), anxiety, and fear about the future increased in reaction to lockdown restrictions imposed by governments (Rubin and Wessely, 2020). During the lockdown period, one of the most important restrictions was distance learning (DL), which forced people to attend classes only on online education and learning platforms. DL compelled a vast number of individuals to adopt information and communication technologies, leading to a significant surge in their development and usage during the lockdown period. DL was instrumental in meeting the dual objective of providing education while ensuring health safety by maintaining physical distancing (“*environmental health*”) (Akuratiya and Meddage, 2020; Toquero, 2020). Most universities and educational institutions worldwide were unprepared for dealing with the transition from face-to-face to distance learning (Crawford et al., 2020). Distance learning was characterized by three dimensions: *social presence*, *social interaction*, and *satisfaction*.

The expression *social presence* refers to the degree of realness assigned to the other in communication (e.g., Short et al., 1976; Kreijns et al., 2014, 2022). *Social presence* includes three groups of characteristics: lesson organization and structure and the methodology or technique used for teaching (e.g., cooperative learning and peer education); clear instructions to help the online students understand the lesson topic; the possibility of using teaching tools and devices for online students (e.g., videos).

*Social interaction* refers to the different forms of communication adopted by users (i.e., students and teachers) when interacting with each other, as well as any other forms of engagement that enable social connections and collaboration in the online learning environment. The quality of social interaction is related to critical thinking and flexibility in the organization of teaching.

*Satisfaction* includes the assessment of individual learning, social presence, and social interaction. All these dimensions enable students’ engagement (Fortune et al., 2011; Gray and DiLoreto, 2016; Bali and Liu, 2018).

Dhawan (2020) identified several strengths and weaknesses associated with DL. The advantages included the ability to offer temporal and spatial flexibility, a vast array of courses and content, and prompt feedback. However, DL also had some drawbacks, such as technical difficulties, challenges in time management, potential distractions, frustrations, anxiety and confusion, and a lack of attentiveness.

Although numerous studies have been conducted on the psychological impact of the COVID-19 pandemic on university students, only a small number of them have specifically investigated the psychological effects resulting from the mandatory use of DL during the COVID-19 lockdown (Cao et al., 2020; Irawan et al., 2020; Russell et al., 2023).

Regarding the relationship between DL and psychological outcomes and characteristics, in the literature, it was evidenced that there is an association between DL and depressive symptoms (Elmer and Stadtfeld, 2020), an increment in stress and a decrease in attention during the online lessons (Quintiliani et al., 2021), an increment of worries about the availability and difficulty in using online platforms (Moawad, 2020) and a larger problem for students in DL to interact with teachers or to find technical support (Attri, 2012; Dodo et al., 2013; Musingafi et al., 2015).

Lastly, cross-sectional and quantitative studies in particular have consistently demonstrated that the COVID-19 pandemic, and the resulting sudden shift to DL, had detrimental impacts on the motivation and mental health of university students. Nicholson et al. (2023) employed a mixed methods design to examine the experiences of students in an Australian University both at the outset of the pandemic in 2020 and again, in a second step at the conclusion of their academic year, 6 months later. Results showed that despite quantitative findings suggesting poorer attitudes toward learning during the pandemic, qualitatively students perceived both positives and negatives towards studying online.

### Instruments to measure students’ perception of face-to-face learning environment

Regards the assessment of students’ perception of face-to-face learning, Mather and Sarkans (2018) used qualitative methodology to collect descriptive data. The instrument assessed learner preference, interactivity, workload, performance, challenges, and preference for future learning. Öhman et al. (2016) developed an instrument to measure Swedish nursing students’ perception of their learning environment. The authors studied the construct validity and reliability of the instrument, which had four factors: supervisor relationship, pedagogical atmosphere, leadership style, and patients’ premises.

Most of the studies in the literature assessed *blended learning*, which is the hybrid learning method that combines face-to-face and online teaching methods (Garrison and Kanuka, 2004; Usta and Özdemir, 2007; Glogowska et al., 2011). Several studies developed instruments to measure students’ perceptions of *blended learning*. Extavour and Allison (2018) administered an online survey at the end of a pharmacy course. The survey was a 5-point Likert-like scale from 1 (Not useful) to 5 (Very useful). Adas and Shmais (2011) developed a 41-item questionnaire to measure students’ perception of *blended learning* in a sample of Palestinian university students. The instrument was a 5-point Likert scale from 1 (Strongly Disagree) to 5 (Strongly Agree) and the self-report assessed three domains: students’ attitudes towards the *blended learning* process; students’ attitudes towards blended learning content; students’ attitudes towards the ease of use of computers. Owston et al. (2013) developed a self-report instrument composed of 31 items, 25 of which were on a 5-point Likert-style scale from 1 (Strongly Disagree) to 5 (Strongly Agree) and six were multiple choice questions in a sample from York University. The instrument was adapted by Bidder et al. (2016) in a sample of Malaysian university students. Ginns and Ellis (2007) developed a self-report instrument to measure students’ perception of *blended learning* in a sample of Australian Veterinary Science students. The instrument was composed of 18 items on a 3-point Likert-style scale (Disagree, Neutral, Agree). The authors studied the factor structure and the reliability of the instrument. Berga et al. (2021) adapted the Web-based Learning Environment Instrument (WBLEI; Chang and Fisher, 2001) to measure nursing students’ perception of online learning environments. The instrument was composed of 37 items on a 5-point Likert-style scale from 1 (Strongly Disagree) to 5 (Strongly Agree). The WBLEI assessed four factors: Interaction, Access, Response, and Results for Maths and Science education students. Studies have analyzed the construct validity, descriptive statistics, and reliability of the instrument (Chang and Fisher, 2001; Berga et al., 2021). These findings had poor generalizability to a greater number of participants, due to administration through the convenience sampling method; only two studies (Ginns and Ellis, 2007; Berga et al., 2021) examined the factor structure of the instruments. Other researchers studied the reliability, frequencies, and descriptive statistics of instruments. Furthermore, instruments we are not suitable for the pandemic context. Finally, studies analyzed perceptions of the difference between face-to-face and distance education or *blended learning* and face-to-face environments (Smith, 2013; Platt et al., 2014; Tichavsky et al., 2015; Wright, 2017; Yilmaz, 2019; Gherheș et al., 2021; Mali and Lim, 2021; Johnson King et al., 2022). These studies were not specifically on measuring students’ perception of face-to-face learning environments.

### Association between self-efficacy, anxiety, and depression in the online and face-to-face learning environments

Few studies have analyzed the association between perceived emotive self-efficacy and clinical variables (e.g., anxiety and depression) in university students (Morales-Rodríguez and Pérez-Mármol, 2019).

Self-efficacy refers to an individual’s belief in his or her capacity to exert control over one’s own motivation, behavior, and social environment and to execute behaviors required to produce specific performance attainments (Bandura and Adams, 1977, Bandura 1986, 1997).

More recently, the concept of self-efficacy has evolved into regulatory, emotional self-efficacy, a process of modulating cognition, emotions, and behavior to achieve goals in life (Caprara et al., 2008). Regulatory, emotional self-efficacy includes two dimensions: self-efficacy in managing negative emotions and self-efficacy in expressing positive emotions (Caprara et al., 2010, 2013). The first dimension allows the modulating of negative emotions (e.g., anger and fear) to control impulsive behaviors; the second dimension allows the expression of positive emotions. Regulatory, emotional self-efficacy enables the promotion of positive emotions (e.g., serenity and happiness), prosocial behavior (e.g., helping and sharing), and academic achievements (Caprara et al., 2011). Positive emotions, academic achievements, and prosocial behaviors increase psychological well-being and decrease depression and anxiety (Mesurado et al., 2018; Picconi et al., 2019). Some studies investigated the association between anxiety, depression, and self-efficacy. Poor self-efficacy generates high levels of worry and rumination which are symptoms of anxiety and depression disorders, respectively (Liu et al., 2019; Zhou and Yu, 2021). It has been noted that challenges in comprehending the significance of emotions can result in individuals amplifying the adverse facets of social scenarios and evading situations that may trigger emotional responses. Difficulties in understanding the role of emotions have been identified, which can lead people to exaggerate negative aspects of social situations and avoid situations that can activate emotional states. In the scholastic context, students with higher scholastic self-efficacy believe that they can achieve their academic goals and, therefore, have higher motivation to study (Zimmerman and Bandura, 1994; Yokoyama, 2019). The beginning of a person’s university career marks the transition from childhood to adulthood and the acquisition of new social roles. These changes can also create higher levels of stress, academic difficulties, and lower levels of well-being. Therefore, it is important to study the role of individual self-efficacy in coping with stressful events (Onyeizugbo, 2011; Arnett et al., 2014; Gutierrez and Park, 2015; Aimé et al., 2017; Faramarzi and Khafri, 2017; Mirzaei-Alavijeh et al., 2017; Grøtan et al., 2019; Morales-Rodríguez and Pérez-Mármol, 2019; Gutiérrez García and Landeros Velázquez, 2020). In particular, some authors found that higher levels of anxiety and depression were associated with poor perceived self-efficacy (Bandura, 1993; Chou, 2018; Zheng et al., 2020). These findings were not confirmed by Faramarzi and Khafri (2017), who did not find any correlations between academic self-efficacy, anxiety, and depression. Their study showed that only alexithymia was negatively correlated with self-efficacy.

Self-efficacy in a DL context is called online learning self-efficacy (OLSE) and is focused on the technological factor of self-efficacy (e.g., internet self-efficacy, learning management self-efficacy, and computer self-efficacy) (Alqurashi, 2019; Aldhahi et al., 2022). OLSE consists of three dimensions: learning-related self-efficacy, technology-related self-efficacy, and time management-related self-efficacy. To the best of our knowledge, only one study has analyzed the association between self-efficacy, anxiety, and depression in the context of DL (Zhou and Yu, 2021). The researchers found a significant, negative association between online learning self-efficacy and anxiety in a sample of Chinese undergraduate students. Furthermore, they found that anxiety mediated the positive association between online self-efficacy and well-being, thus reducing self-efficacy.

### Students’ perception of online learning during the COVID-19 pandemic

Several studies examined students’ perceptions of online learning during the COVID-19 pandemic. In particular, some authors (Almusharraf and Khahro, 2020; Mukhtar et al., 2020; Bączek et al., 2021) found a high level of satisfaction regarding DL in a sample of post-secondary and university students. Data showed that digital support (e.g., slides, audio, and video), teaching modes (e.g., group or individual discussions), the possibility of studying according to own personal pace, and the possibility of staying at home made lessons more accessible and understandable. Furthermore, the majority of the students worked towards their academic goals. A study on a sample of dental and medical students in Pakistan showed the positive aspects of DL: free access to online materials and a greater sharing of videos made by teachers with laboratory and clinical expertise (Mukhtar et al., 2020).

Several studies (Agung et al., 2020; Bahasoan et al., 2020; Hamid et al., 2020; Mukhtar et al., 2020; Nambiar, 2020; Lemay et al., 2021) explored the unfavorable facets of DL, encompassing social and psychological issues as well as technological obstacles. With regard to social and psychological difficulties, the majority of students complained of social isolation, an increase in stress and anxiety, difficulty in understanding learning content, difficulty in maintaining high concentration levels during lessons, a decrease in motivation, and poor perceived emotional and scholastic self-efficacy. Mukhtar et al. (2020) reported that dental and medical students expressed concerns regarding the absence of opportunities to interact with patients.

Nicholson et al.’s (2023) qualitative results further highlighted that the DL experience is not the same for everyone and suggests the need to reconsider the standard approaches to providing support to students. In each case, students reported poor mental health and low levels of commitment and motivation, also expressing their need for contact with peers.

Evidence suggests that students’ achievement emotions are important contributors to their learning and success online. It is, therefore, essential to understand and support students’ emotional experiences in order to enhance online education, especially in the COVID-19 context. However, to date, very few studies (Shao et al., 2023) have investigated how students’ achievement emotions may be affected by teaching and learning factors in online learning environments.

Technical problems (e.g., outages of internet connection) involved the difficulty of getting an internet connection in their homes and the scarce availability of digital support (e.g., CDs, pen drives, and memory cards), the inefficacy of online courses due to high costs, the higher difficulty in completing course work and signal instability during online lessons. These studies did not examine the nomological network between students’ perception of online learning, anxiety, depression, and perceived emotional and scholastic self-efficacy during the COVID-19 pandemic. Furthermore, these studies examined students’ perceptions of online learning during the COVID-19 pandemic without psychometric tests.

### Aims of the study

The principal aim of this study was the creation and validation of a new questionnaire to measure students’ perceived quality of DL and an analysis of the nomological structure of individual socio-demographic characteristics, anxiety, depression, and emotional and scholastic perceived self-efficacy relating to DL.

In relation to the nomological structure, we developed the following hypotheses:

1. H1: negative variations of behavior and life habits are significantly and negatively correlated with students’ perceived quality of DL;
2. H2: anxiety has a negative connection with students’ perceived quality of DL;
3. H3: depression has a negative connection with students’ perceived quality of DL;
4. H4: perceived emotional self-efficacy has a positive connection with students’ perceived quality of DL;
5. H5: perceived scholastic self-efficacy has a positive connection with students’ perceived quality of DL.

## Materials and methods

### Data collection and procedures

A total of 429 participants responded when contacted. Regarding the bias of response in the Perceived Quality of Distance Learning questionnaire, women tended to choose higher response categories (from partially agree to strongly agree) for items 1 (“I can easily access the internet”), 3 (“I own computer devices to attend classes”), 6 (“I think distance learning offers better study organization for working students”), and 10 (“I can record lessons”), and lower response categories (from strongly disagree to partially disagree) for items 2 (“I feel detached from physical and group relationships”), 8 (“I feel fatigue, have eyestrain, and headaches”), and 19 (“I feel nostalgic recalling pre-COVID-19 events of physical and social interaction”). Men tended to choose higher response categories for items 1 (“I can easily access the internet”), 3 (“I own computer devices to attend classes”), 17 (“I feel embarrassed during written exams”), and 20 (“I cry more easily when I think about friends I have not seen in a long time due to pandemic restrictions”).

There were no refusals regarding the completion of the questionnaire. In fact, all students gave their consent to respond to all items.

The snowball sampling method was used, i.e., the authors asked their students to distribute the link among their friends. The sample was obtained through quota sampling, as individuals were selected from pre-established groups (university courses and training programs with a requirement of having attended online classes). Therefore, the representativeness of the sample cannot be guaranteed. The study herein was conducted according to the principles of the Helsinki Declaration^1^ (Accessed on 22 August 2021), APA Ethics Code, and European and Italian Privacy Law (i.e., EU Reg. 679/2016, GDPR and Legislative Decree no. 196/2003, namely the Personal Data Protection Code). It was approved by the Psychology, Communication, and Social Sciences PhD curriculum meeting, (University of Macerata. Prot. no. 0041598 of 31/03/2021 - UOR: SI000018 - Classif. VI/6).

The test battery was administered online and was created using Google Forms. The link to the survey was distributed via WhatsApp and the platforms used during DL (e.g., Microsoft Teams, Google Meet, and Zoom).

The instrument can be requested from Dr. Maria Rita Sergi (mariaritasergi@libero.it).

### Participants

In total, 429 (74.8% female) participants were contacted for the collection of data. The mean age was 23.20 years (SD = 5.91). Participants were students of different categories: 219 (51.0%) were university students attending lessons in the humanities faculty, 158 (36.8%) were university students attending lessons in scientific faculties, 16 (3.7%) were university students attending linguistic faculties, 15 (3.5%) were students attending non-university training courses, 7 (1.6%) were students attending economic faculties, 6 (1.4%) were students attending law faculties; 4 (0.9%) were students attending PhD and masters courses, and 4 (0.9%) were students who did not indicate their faculties. Furthermore, 203 participants (47.3%) lived in Central Italy, 194 lived in Southern Italy (45.2%), 28 (6.5%) lived in Northern Italy, and 4 (0.9%) lived on the Italian islands (i.e., Sicily and Sardinia).

### Measures

#### Socio-demographic characteristics

Socio-demographic characteristics were age, educational level, and behavioral changes as a result of the COVID-19 pandemic.

#### Anxiety

The state of anxiety was measured with the State–Trait Anxiety Inventory, X1 form (STAI-X1; Spielberg et al., 1970). STAI-X1 is a 20-item self-report inventory and responses to each item are given using a 4-point scale ranging from 1 (“none”) to 4 (“very much”) and was presented at the beginning of the survey (*α* = 0.95). A reduced form of STAI-X1 (STAI-X1/R) with 10 items, was presented at the end of the survey (*α* = 0.94). The comparison between the STAI-X1 and the STAI-X1/R score was used to evaluate whether the level of anxiety decreased or increased while completing the survey. Trait anxiety was assessed with the State–Trait Anxiety Inventory, X2 form (STAI-X2; Spielberg et al., 1970) which consists of 20 items. Responses in each item were given using a 4-point scale ranging from 1 (“hardly ever”) to 4 (“almost always”) (*α* = 0.92).

#### Depression

The cognitive and somatic factors of depression (e.g., psychomotor slowdown and sadness) were measured with the Depression Questionnaire (QD; Bertolotti et al., 1997), comprising 24 items. Each item had a dichotomic scale (yes vs. no) (KR20 = 0.865).

#### Perceived self-efficacy

Perceived Emotive Self-Efficacy (PESE; Caprara, 2001) is a scale for assessing the ability to express positive emotions (seven items) (*α* = 0.90) and to manage negative emotions (eight items) (*α* = 0.875) on a 5-point Likert scale. The higher the score, the higher the ability to express positive emotions or manage negative emotions. Scholastic Perceived Self-Efficacy (SPSE; Pastorelli and Picconi, 2001) is a one-dimensional scale consisting of nine items, on a 5-point Likert scale. This scale assessed students’ beliefs in achieving scholastic objectives (*α* = 0.90).

#### Perceived quality of distance learning questionnaire

Perceived quality of distance learning (PQDL) is a questionnaire for assessing students’ perception of DL quality. PQDL consists of 32 items that assessed students’ perceived quality of DL. Items were determined through interviews. In particular, the questionnaire assesses students’ ability to use online platforms and applications, organize their online lessons, cope with technical difficulties, attention and concentration during online lessons, and their cognitive and emotional reactions to DL (worries and irrational thoughts about the pandemic). Participants responded using a 5-point Likert scale ranging from 1 (“I strongly disagree”) to 5 (“I strongly agree”). The higher the score, the higher the perceived quality of DL. Examples of items were: “*I have the possibility to record lessons*,” “*Despite restrictions, I still have hope for the future*,” “*I face exam sessions with more motivation*.” The instrument was developed on the basis of previous literature (Wagner, 1994; Huang, 2002; Wang et al., 2014; Martin and Rimm-Kaufman, 2015) and experiences of research in direct contact with students. Previous literature analyzed connectivism as a new learning theory based on the digital age (Siemens, 2004; Goldie, 2016). In particular, connectivism is defined as *actionable knowledge*, in which new connections, deriving from various information sources (such as computers network and the Web), are established and integrated in users’ minds. This process leads one to critically evaluate situations and contexts and increases critical thinking skills (Duke et al., 2013). According to this point of view, connectivism can be considered a new theory of mind, because actionable knowledge builds new neural connections (Goldie, 2016). In addition, connectivism is evaluated in distance learning, which is characterized as collaborative learning. It is defined by an open, extended interaction, in which “connections are extended from individuals to groups, from small groups to massive possibilities” (Wang et al., 2014, p. 125).

### Statistical analyses

#### Descriptive statistics

Descriptive statistics for the collected data were means, standard deviations, skewness, and kurtosis. Skewness and kurtosis values between-2 and 2 indicate a normal distribution of the data (Gravetter and Wallnau, 2014).

#### Exploratory factor analysis

To assess the factor structure of the PQDL questionnaire on students’ perception of DL, the sample was randomly divided into two subsamples (Bollen, 1986). The factorial structure of the instrument in the first subsample was analyzed with an Exploratory Factor Analysis (EFA), while the data from the second subsample were analyzed with a Confirmatory Factor Analysis (CFA). Regarding the EFA, the principal axis factoring method of extraction was used. The number of latent factors was chosen on the basis of a scree plot (Cattell, 1966) and eigenvalues >1 (Kaiser, 1974). The promax rotation was used for oblique factor rotation. In addition, all items with factor loadings < ǀ.30ǀ or with loadings > ǀ.30ǀ in two or more factors were deleted.

The multidimensional scaling plot was used to visualize the matrix of distance among variables, using the PROXSCAL algorithm (Busing et al., 1997).

#### Confirmatory factor analysis

All CFAs were performed using the “Maximum Likelihood” estimation method (Muthen and Kaplan, 1985). Goodness-of-fit indices were the χ^2^, the root mean square error of approximation (RMSEA) and the corresponding confidence interval (90% RMSEA), the Comparative Fit Index (CFI), the Tucker-Lewis fit Index (TLI), and Akaike’s information criterion (AIC). Models with an acceptable fit should have an RMSEA <0.08, and CFI and TLI >0.90 (Hu and Bentler, 1999; Schermelleh-Engel et al., 2003). AIC allows the comparison of two or more factorial models. The best model must have smaller AIC values (Hu and Bentler, 1999). Mardia’s normality test is used to assess the normality of data distribution. A low Mardia’s value indicates the normality of data distribution (Mardia’s normalized estimate = 0.534; Mardia, 1974). To reduce factorial structure complexity, the items of the scale were grouped into parcels. This technique is based on the grouping of many variables into fewer groups or levels (parcels) that have greater reliability in relation to single items (Bandalos, 2002; Nasser-Abu Alhija and Wisenbaker, 2006; Saggino et al., 2015).

#### Reliability

The reliability of the PQDL questionnaire was calculated with Cronbach’s Alpha (Cronbach, 1960) and McDonald’s Omega (Zinbarg et al., 2005; Dunn et al., 2014). Values >0.90 indicate excellent reliability, values between 0.80 and 0.90 indicate good reliability, values between 0.70 and 0.80 indicate discrete reliability, values between 0.60 and 0.70 indicate sufficient reliability, and values <0.60 indicate inadequate reliability (Balsamo, 2017).

#### Nomological analysis

The convergent validity of the PQDL questionnaire on students’ perception was assessed with bivariate correlations between the PQDL scores and scores of self-efficacy. Divergent validity was assessed with bivariate correlations between the PQDL scores and scores of anxiety and depression. In addition, bivariate correlations between the PQDL scores and socio-demographic characteristics were calculated. Educational level was transformed into dummy variables with values “0″ (students attending non-university training courses) and “1″ (university students). Also, behavioral changes (sleep problems, change in life habits, eating disorders, reduction of appetite, and sedentary life) due to pandemic restrictions were transformed into variables with dummy coding values “0″ (no or negligible change) and “1″ (moderate or strong change).

#### Predictive validity

Multiple regressions were performed to analyze the predictive validity of socio-demographic and psychological factors on PQDL questionnaire scores.

All statistical analyses were made using SPSS 25.0 (IBM Corp., 2017) for Windows. McDonald’s Omegas were estimated using JASP Version 0.11.1.0 (JASP Team, 2019). EFA and CFA were carried out using Mplus 7.0 (Muthén and Muthén, 1998–2012).

## Results

The majority of participants were university students and most of the sample suffered from sleep problems and changes in life habits due to the pandemic (Table 1).

**Table 1:** Characteristics of participants (*N* = 429).

Educational level	Frequency (*f*)	Percentage (%)
University students	406	94.5
Students attending non-university training courses	15	3.5%
PhD/Master	4	0.9%
Missing	4	0.9%
Behavioral changes	Frequency (*f*)	Percentage (%)
Sleep problem	236	55.05%
Change of life habits	95	22.1%
Eating disorders	67	15.65%
Reduction of appetite	27	6.3%
Sedentary life	4	0.9%

Table 2 shows the descriptive statistics of all items of the PQDL questionnaire on students’ perceived quality of DL. The skewness and kurtosis values are all between –2 and 2.

**Table 2:** Mean, standard deviation and normality indices of items of the perceived quality of distance learning questionnaire (*N* = 429).

PQDL items	Mean	SD	Skewness	Kurtosis
(1) I can easily access the internet.	4.16	1.033	−1.200	0.765
(2) I feel detached from physical and group relationships.	2.16	1.181	0.876	−0.104
(3) I own computer devices (e.g., tablet, PC, etc.) to attend classes.	4.67	0.628	−1.709	1.608
(4) I perceive greater communicative contact between teachers and students.	2.61	1.188	0.181	−0.901
(5) There is a reduction in expenses for commuter and/or off-site students.	3.64	1.371	−0.660	−0.770
(6) I think distance learning offers better study organization for working students.	3.90	1.206	−0.946	−0.019
(7) During my classes, I can stay focused for several hours.	2.42	1.265	0.387	−1.072
(8) I feel fatigue, have eyestrain, and headaches.	2.18	1.231	0.891	−0.175
(9) I feel more engaged with the educational topics proposed in class.	2.83	1.167	−0.011	−0.847
(10) I can record lessons.	3.85	1.213	−0.854	−0.231
(11) I share lecture topics with other students.	3.38	1.220	−0.365	−0.807
(12) I perceive a better quality in my learning.	2.78	1.222	0.158	−0.817
(13) I can better organize my study material.	3.29	1.260	−0.208	−0.960
(14) During class hours, it is easier for me to divert my attention from my current worries.	4.16	1.033	−0.037	−0.957
(15) I perceive greater difficulty in taking oral exams, due to the difficulty of physical contact between teachers and students.	3.22	1.380	−0.184	−1.164
(16) Despite the restrictions, I am hopeful about the future.	3.60	1.183	−0.521	−0.633
(17) I feel embarrassed during written exams.	3.97	1.234	−0.974	−0.153
(18) I am concerned about my academic commitments, as they may change due to new government restrictions.	2.94	1.354	0.156	−1.130
(19) I feel nostalgic recalling pre-COVID-19 events of physical and social interaction.	2.07	1.316	0.986	−0.293
(20) I cry more easily when I think about friends I have not seen in a long time due to pandemic restrictions.	3.25	1.480	−0.213	−1.361
(21) I feel that I study with “greater effort.”	2.69	1.401	0.308	−1.160
(22) I procrastinate the start of my daily study sessions, due to negative thoughts about the consequences of the pandemic.	3.45	1.403	−0.427	−1.122
(23) I think of the moment of social and economic uncertainty with pessimism.	2.84	1.263	0.275	−0.913
(24) Being informed about the health and social situation is not an obstacle in pursuing my established educational objectives.	3.41	1.209	−0.395	−0.709
(25) I feel ‘lost’ in this pandemic moment and this makes concentration on studying difficult.	2.99	1.428	0.074	−1.326
(26) Despite the precariousness caused by the pandemic, I have faith in the economic, social, and job recovery.	3.14	1.157	−0.107	−0.828
(27) Thinking about the moment when I will be able to attend classes in person makes studying easier for me.	3.31	1.192	−0.129	−0.862
(28) I feel uneasy dealing with distance learning.	3.67	1.258	−0.469	−0.953
(29) I have the tenacity to stick to the syllabus and educational programs, despite the restrictions imposed by the government.	3.31	1.192	−0.206	−0.857
(30) I face exam sessions with more motivation.	2.58	1.208	0.386	−0.687
(31) I perceive greater difficulty in taking written exams, due to the difficulty of physical contact between teachers and students.	3.68	1.333	−0.682	−0.717
(32) I feel ashamed during oral exams.	3.02	1.445	0.042	−1.326

Reverse items: 8, 15, 17, 18, 19, 29, 21, 22, 23, 25, 27, 28, 31, 32.

### Exploratory factor analysis and reliability

**Figure 1:**
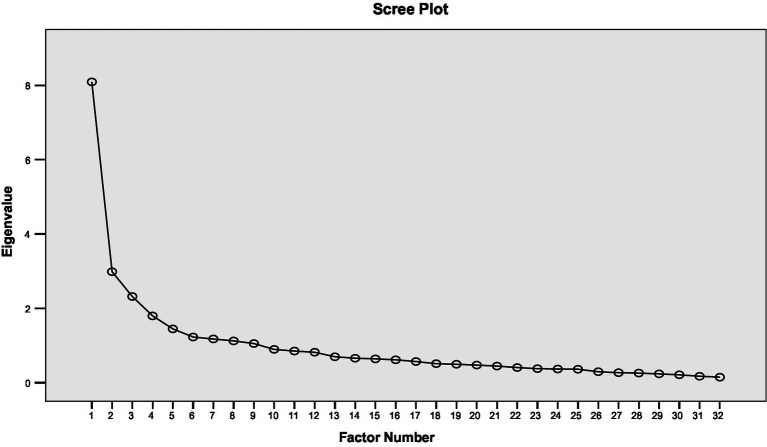
Scree plot of EFA (*N* = 214).

EFA was performed on a sample of 214 participants. Bartlett’s Test of Sphericity [χ^2^ (df = 496) = 2940.499; *p* < 0.001] and Kaiser–Meyer–Olkin (KMO = 0.849) confirmed sample adequacy for factor analysis. Initial eigenvalues were: 7.695; 2.522; 1.882 and 1.348. Figure 1 shows the scree plot. By combining the scree plot and eigenvalues, the two-factor solution seemed the most probable structure of the scale.

**Figure 2:**
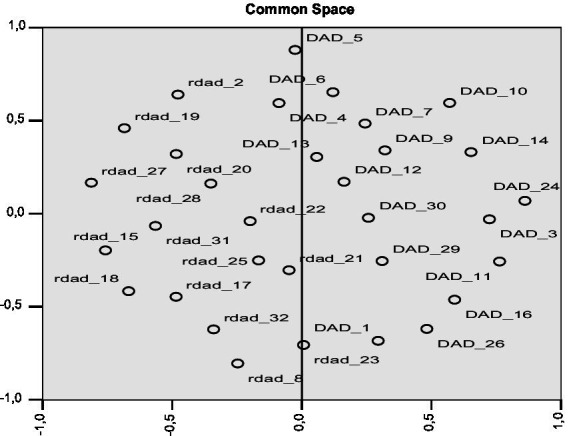
Multidimensional scaling plot (*N* = 214).

Items 1-3-2-5-11-16-24-26-27 were removed for having loadings < ǀ.30ǀ or double-factor loadings. The first factor accounts for 30.20% of the total variance and the second factor for 8.84% of the total variance. The 2-factor solution explained 39.04% of the variance (Table 3). Based on the content items, Factor 1 was named Distance Learning Organization (DLO) and Factor 2 was named Cognitive-Emotive Reaction to DL (CER-DL). Both DLO and CER-DL had a good level of reliability (*α* = 0.86 and α = 0.865, respectively, and ω = 0.87 and ω = 0.87, respectively). Figure 2 shows the matrix of distance among variables as items are distributed between two dimensions.

**Table 3:** Item loadings and communalities (*h*^2^) of the perceived quality of distance learning questionnaire (*N* = 214).

PQDL items	Factor 1	Factor 2	*h* ^2^
(1) I can easily access the internet.	0.287	0.040	0.097
(2) I feel detached from physical and group relationships.	0.082	0.283	0.113
(3) I own computer devices (e.g., tablet, PC, etc.) to attend classes.	0.299	−0.054	−0.159
(4) I perceive greater communicative contact between teachers and students.	**0.626**	−0.092	0.336
(5) There is a reduction in expenses for commuter and/or off-site students.	0.262	−0.046	−0.139
(6) I think distance learning offers better study organization for working students.	**0.675**	−0.071	0.407
(7) During my classes, I can stay focused for several hours.	**0.604**	0.095	0.438
(8) I feel fatigue, have eyestrain, and headaches.	−0.195	**0.494**	0.175
(9) I feel more engaged with the educational topics proposed in class.	**0.896**	−0.163	0.667
(10) I can record lessons.	**0.343**	−0.057	0.099
(11) I share lecture topics with other students.	0.629	−0.365	−0.457
(12) I perceive a better quality in my learning.	**0.848**	0.049	0.768
(13) I can better organize my study material.	**0.847**	−0.033	0.688
(14) During class hours, it is easier for me to divert my attention from my current worries.	**0.504**	−0.120	0.201
(15) I perceive greater difficulty in taking oral exams, due to the difficulty of physical contact between teachers and students.	0.039	**0.394**	0.173
(16) Despite the restrictions, I am hopeful about the future.	0.255	0.093	−0.061
(17) I feel embarrassed during written exams.	0.070	**0.361**	0.163
(18) I am concerned about my academic commitments, as they may change due to new government restrictions.	−0.114	**0.522**	0.219
(19) I feel nostalgic recalling pre-COVID-19 events of physical and social interaction.	−0.007	**0.363**	0.129
(20) I cry more easily when I think about friends I have not seen in a long time due to pandemic restrictions.	−0.022	**0.616**	0.365
(21) I feel that I study with “greater effort.”	0.157	**0.691**	0.622
(22) I procrastinate the start of my daily study sessions, due to negative thoughts about the consequences of the pandemic.	0.131	**0.698**	0.606
(23) I think of the moment of social and economic uncertainty with pessimism.	−0.270	**0.737**	0.394
(24) Being informed about the health and social situation is not an obstacle in pursuing my established educational objectives.	0.290	−0.112	0.059
(25) I feel ‘lost’ in this pandemic moment and this makes concentration on studying difficult.	0.012	**0.807**	0.662
(26) Despite the precariousness caused by the pandemic, I have faith in the economic, social, and job recovery.	0.275	0.064	−0.091
(27) Thinking about the moment when I will be able to attend classes in person makes studying easier for me.	0.034	0.273	0.086
(28) I feel uneasy dealing with distance learning.	0.205	**0.541**	0.459
(29) I have the tenacity to stick to the syllabus and educational programs, despite the restrictions imposed by the government.	**0.464**	0.204	0.363
(30) I face exam sessions with more motivation.	**0.662**	0.108	0.530
(31) I perceive greater difficulty in taking written exams, due to the difficulty of physical contact between teachers and students.	0.121	**0.457**	0.285
(32) I feel ashamed during oral exams.	−0.043	**0.502**	0.230
% of variance	30.204	8.841	
Cumulative %	30.204	39.045	

Significant factor loadings (> |0.30|) are in bold types. Reverse items: 8, 15, 17, 18, 19, 29, 21, 22, 23, 25, 27, 28, 31, 32.

### Confirmatory factor analysis

CFA was performed on 215 participants. Partly considering the results obtained with EFA and partly on the basis of a theoretical approach, we hypothesized four-factor structures for the PQDL scale: a model with only one first-order latent variable (M1), a model with two correlated first-order latent variables (M2), a model with two uncorrelated first-order latent variables (M3), and a hierarchical model with first-and secondary-order latent variables (M4). The PQDL items were grouped into six parcels (f1p1, f1p2, f1p3, f2p2, f2p1, f2p3). Parceling reduces the magnitude of specific variances that lead to correlated residuals and dual-factor loadings in a given model (Little et al., 2013). In the M1 model, all parcels loaded on the single first-order factor; in M2, f1p1, f1p2, and f1p3, which grouped the items of the DLO subscale, loaded on one first-order factor and f2p2, f2p1, and f2p3, which grouped the items of the CER-DL subscale, loaded on the other first-order factor. In M2, both factors were uncorrelated. M3 had the same structure as M2, with the only exception that both factors were correlated. M4 had the same structure as M2, with the exception that both first-order factors loaded on a single second-order general factor. Table 4 shows that M2 is the model with the best fitting. DLO and CER-DL were correlated.

**Table 4:** Goodness-of fit indexes of the perceived quality of distance learning questionnaire (*N* = 215).

Model	χ^2^	df	RMSEA	CFI	TLI	SRMR	Interval RMSEA	AIC
M1	238.603	9	0.344	0.576	0.293	0.177	0.307 0.383	3148.017
M2	19.605	8	0.082	0.979	0.960	0.040	0.036 0.129	2931.020
M3	32.374	9	0.110	0.957	0.928	0.113	0.071 0.152	2941.789
M4	19.624	7	0.092	0.977	0.950	0.041	0.045 0.141	2933.039

One factor model (M1), two correlated first-order factors model (M2), two independent factors model (M3), hierarchical model (M4). df, degrees of freedom; RMSEA, root-mean-square error of approximation; CFI, comparative fit index; TLI, Tucker–Lewis fit index; SRMRS, standardized root-mean square residual; AIC, Akaike information criterion.

### Association between distance learning, socio-demographic variables anxiety, depression, and perceived self-efficacy

Table 5 shows significant correlations between DLO, CER-DL, emotional management of scholastic achievements, and the socio-demographic variables anxiety, depression, and perceived self-efficacy. Correlations ranged from *r* = −0.160; *p* < 0.01 between DLO and Behavioural changes to *r* = −0.435; p < 0.01 between CER-DL and STAI-X1.

**Table 5:** Zero-order correlation analysis among factors of the perceived quality of distance learning questionnaire, socio-demographic variables, state anxiety and trait anxiety, depression, and perceived self-efficacy (*N* = 429).

Factors of the questionnaire on students’ perception of online classes	Age	Educational level	Behavioral changes	STAI-X1	STAI-X2	STAI-X1/R	DQ	Perceived self-efficacy of negative emotions	Perceived self-efficacy of positive emotions	Scholastic perceived self-efficacy
Distance learning organization	0.198**	−0.038	−0.160**	−0.277**	−0.279**	−0.258**	−0.335**	0.232**	0.043	0.384**
Cognitive-emotive reaction	0.222**	−0.058	−0.325**	−0.435**	−0.429**	−0.411**	−0.468**	0.242**	−0.045	0.163**

p, significance; **p < 0.01. State Anxiety Inventory (STAI-X1) at the beginning of the survey; State–Trait Anxiety Inventory Reduced Form (STAI-X1/R) at the end of the survey; Trait Anxiety Inventory (STAI-X2); Depression Questionnaire (DQ).

### Questionnaire predictors on perceived quality of distance learning questionnaire score

#### Predictive validity

Regression analyses were used to assess the predictive validity of age, behavioral changes, state of anxiety at the beginning of the survey and at the end of the survey, trait anxiety, depression, Perceived Self-Efficacy of negative emotions, and Perceived School Self-Efficacy on the DLO and CER-DL. Results showed that age (*t* = 4.394), depression (*t* = −3.008), and Perceived School Self-Efficacy (*t* = 6.842) were significant predictors of DLO. Age (*t* = 4.505), behavioral changes (*t* = −2.451), and depression (*t* = −2.845) were significant predictors of CER-DL (Table 6).

**Table 6:** Regression analysis for assessing predictive validity of age, behavioral changes, state anxiety at the beginning of the survey and at the end of the survey; trait anxiety, depression, perceived self-efficacy of negative emotions and perceived school self-efficacy on DLO and CER-DL.

Distance learning organization										
Predictors	*β*	β*	*t*	*p*(*t*)	VIF	*p*(η^2^)	*R* ^2^	*F*(df1,df2)	*p*(F)	*f* ^2^
Age	0.026	0.192	4.394	**0.000**	1.046	0.035	0.055	16.205 (8,420)	<0.001	0.05
Behavioral changes	0.018	0.010	0.196	0.845	1.301	0.000				
STAI-X1	−0.007	−0.109	−1.345	0.179	3.590	0.003				
STAI-X2	0.008	0.124	1.498	0.135	3.761	0.004				
STAI-X1/R	−0.001	−0.011	−0.151	0.880	3.089	0.000				
DQ	−0.037	−0.233	−3.008	0.**003**	3.286	0.016				
Perceived self-efficacy of negative emotions	−0.002	−0.015	−0.267	0.790	1.748	0.000				
Scholastic perceived self-efficacy	0.038	0.330	6.842	**0.000**	1.275	0.085				

Cognitive-emotive reaction to distance learning										
Predictors	*β*	β*	*t*	*p*(t)	VIF	*p*(η^2^)	*R* ^2^	*F*(df1,df2)	*p*(F)	*f* ^2^
Age	0.027	0.189	4.505	**0.000**	1.046	0.034	0.084	21.563 (8,420)	<0.001	0.08
Behavioral changes	−230	−0.115	−2.451	**0.015**	1.301	0.010				
STAI-X1	−0.009	−0.137	−1.762	0.079	3.590	0.005				
STAI-X2	−0.007	−0.100	−1.259	0.209	3.761	0.002				
STAI-X1/R	−0.007	−0.062	−0.855	0.393	3.089	0.001				
DQ	−0.034	−0.212	−2.845	**0.005**	3.286	0.013				
Perceived self-efficacy of negative emotions	−0.010	−0.073	−1.340	0.181	1.748	0.003				
Scholastic perceived self-efficacy	0.002	0.014	0.297	0.766	1.275	0.000				

Significative values are in bold types State Anxiety Inventory (STAI-X1) at the beginning of the survey; State–Trait Anxiety Inventory Reduced Form (STAI-X1/R) at the end of the survey; Trait Anxiety Inventory (STAI-X2); Depression Questionnaire (QD). β, raw beta coefficients; β*, standardized beta coefficients; VIF, variance inflation factor; *p*(η^2^), partial eta squared. *R*^2^, multiple correlation coefficient. df1 and df2, degrees of freedom for numerator and denominator, respectively. *f*^2^, effect size for multiple regression.

## Discussions

The principal aim of this study was the creation and validation of a new questionnaire to measure students’ perceived quality of DL in a sample of Italian university students and Italian students attending non-university training courses. To date, no specific psychometric tools have been available to assess students’ reactions to online lessons during the COVID-19 pandemic.

Furthermore, the literature has not studied the nomological network between students’ perception of online learning, anxiety, depression, and perceived emotional and scholastic self-efficacy during the pandemic.

### The construction of a new instrument to assess the perceived quality of distance learning

The new instrument for measuring students’ perception of online lessons, the Perceived Quality of Distance Learning (PQDL), has shown good psychometric reliability. The PQDL consists of two subscales: the Distance Learning Organization (DLO) subscale and the Cognitive-Emotive Reaction to Distance Learning (CER-DL). The first subscale measures the perceived organization of DL. In particular, DLO assessed the quality of the online interaction between students and teachers, the possibility of recording lessons, the perception of DL quality, the organization of online lessons, and the control of sustained attention during lessons. The second subscale assessed the cognitive and emotive reactions to DL. In particular, the CER-DL subscales assessed the sensation of embarrassment during online examinations, individual engagement in DL, emotional instability, the tendency to procrastinate with online lessons, irrational thoughts regarding the pandemic, and individual pessimism. Procrastination allows a person to avoid or postpone difficult and frustrating situations. Furthermore, procrastination is linked with cognitive processes of worry and rumination (Constantin et al., 2018; Gautam et al., 2019; Mohammadi Bytamar et al., 2020). Worry involves thinking about potential future threats or negative events that may happen. Rumination, on the other hand, involves a persistent focus on past events or mistakes, often accompanied by feelings of guilt or regret (Ellis et al., 2010). Our instrument lies within the growing field of research that explores online learning, showing two principal dimensions: the use of digital skills and cognitive schemas related to the pandemic. Indeed, online learning might help to improve digital skills, such as writing emails or using touch screens (Jackman et al., 2021). Cognitive schemas are a representation of events, that become dysfunctional when thinking errors occur (*beliefs*). Beliefs are reflected in words (such as *must* or *it’s terrible*) connected to the consequences of the pandemic (Beck and Haigh, 2014).

Although there are many studies about individual reactions to DL in the literature (Agung et al., 2020; Almusharraf and Khahro, 2020; Bahasoan et al., 2020; Hamid et al., 2020; Mukhtar et al., 2020; Nambiar, 2020; Bączek et al., 2021; Lemay et al., 2021), our study is the first that attempts to assess this reaction using a questionnaire completely dedicated to perceived quality of DL during the pandemic.

### Hypothesis testing

In relation to our principal hypotheses, hypothesis H1 was confirmed: the changes in behavior habits and styles due to the lockdown (sleep problems, changes in life habits, eating disorders, reduction of appetite, and sedentary life) predicted low score levels in CER-DL.

Hypotheses H2 and H3 were verified: lower levels of anxiety and depression were correlated with students’ better perceived quality of DL. In addition, lower levels of depression predicted higher scores of DLO and CER-DL.

Hypothesis H4 was partially verified. Only the perceived emotional self-efficacy of negative emotions showed a positive correlation with students’ perceived quality of DL. Finally, hypothesis H5 was verified. High Perceived School Self-Efficacy predicted high DLO scores.

Our study confirmed the presence of significant correlations between anxiety, depression, and perceived self-efficacy (Aimé et al., 2017; Mirzaei-Alavijeh et al., 2017; Chou, 2018; Alqurashi, 2019; Grøtan et al., 2019; Morales-Rodríguez and Pérez-Mármol, 2019; Gutiérrez García and Landeros Velázquez, 2020; Zheng et al., 2020; Zhou and Yu, 2021; Aldhahi et al., 2022). Our data showed that a high score in DLO and CER-LS, in other words, a high individual ability in organizing DL and a positive reaction to online lessons, respectively, were significantly related to low levels of depression and high levels of perceived scholastic self-efficacy.

### Limitations

The principal limitations of our study were that participants were not subjects with severe mental illness and that the sample was mainly composed of university students. In the future, we hope to replicate the study with a sample extracted from the clinical population (people with severe anxiety and depression problems) and with a sample of non-university students (for example primary and secondary school students).

## Conclusion

The aim of our study was the construction and development of a new psychometric tool to assess students’ reactions to DL. The new instrument, the Perceived Quality of Distance Learning, consists of two subscales: Distance Learning Organization and Cognitive-Emotive Reaction to Distance Learning. The dimensions assessed by these subscales were both predicted by depression, while the Perceived School Self-Efficacy only predicted DLO scores and behavioral changes only predicted CER-DL scores.

### Practical implications

Shared activities to reduce social isolation and improve the planning of DL could reduce depression and increase perceived self-efficacy. In particular, future educational programs should consider the social impact of DL. Good planning of distance learning helps students and teachers to organize online lessons and courses (the educational methodology or technique used, e.g., cooperative learning or peer education) with less emotional impact. Finally, better communication strategies between students and teachers in online lessons would improve the quality of DL. All these interventions could reduce the level of depression and increase the level of perceived self-efficacy in students using DL.

### Theoretical implications

According to Learning Theories for Online Education, Learning Experience is characterized by three components: Social presence, Teaching Presence, and Cognitive Presence (Picciano, 2021). These components are integrated into interactions between students and teachers (Anderson, 2001). According to Connectivism (Siemens, 2004), learning is transformed from an individual perspective to the shared exchange of information within a group through communication networks (Siemens, 2004). Online Collaborative Learning (Harasim, 2012) emphasizes the significance of interaction between students and teachers, who act as facilitators of knowledge building.

## Data availability statement

The raw data supporting the conclusions of this article will be made available by the authors, without undue reservation.

## Ethics statement

The studies involving human participants were reviewed and approved by University of Macerata, Italy. The patients/participants provided their written informed consent to participate in this study.

## Author contributions

MS assisted with the design of the study, assisted with the data analyses, recruited the sample, wrote the manuscript, and collaborated in editing the final manuscript. LP designed the study, collaborated in recruiting the sample, collaborated in writing the manuscript, and collaborated in editing the final manuscript. AS assisted with the design of the study, collaborated in the data analyses, and collaborated in writing the manuscript. AF designed the study, collaborated in recruiting the sample, and collaborated in editing the final manuscript. RB collaborated in recruiting the sample and collaborated in editing the final manuscript. MT assisted with the design of the study, collaborated in writing the manuscript, and collaborated in editing the final manuscript. All authors contributed to the article and approved the submitted version.

## Conflict of interest

The authors declare that the research was conducted in the absence of any commercial or financial relationships that could be construed as a potential conflict of interest.

## Publisher’s note

All claims expressed in this article are solely those of the authors and do not necessarily represent those of their affiliated organizations, or those of the publisher, the editors and the reviewers. Any product that may be evaluated in this article, or claim that may be made by its manufacturer, is not guaranteed or endorsed by the publisher.

## Footnotes

^1^ https://www.wma.net/what-we-do/medical-ethics/declaration-of-helsinki/
